# Personal Time, Parental Fairness, School Adjustment and Physical Activity Levels as Indicators of Executive Functions in Children and Adolescents

**DOI:** 10.3390/bs16060941

**Published:** 2026-06-08

**Authors:** Felipe Caamaño-Navarrete, Carlos Arriagada-Hernández, Lorena Jara-Tomckowiack, Guido Contreras-Diaz, Cristian Álvarez, Claudio Hernández-Mosqueira, Carla Figueroa-Saavedra, Roberto Lagos-Hernández, Gerardo Fuentes-Vilugrón, Pedro Delgado-Floody

**Affiliations:** 1 Physical Education Career, Universidad Autónoma de Chile, Temuco 4780000, Chile; felipe.caamano@uautonoma.cl (F.C.-N.); roberto.lagos@uautonoma.cl (R.L.-H.);; 2 Facultad de Educación, Universidad Católica de Temuco, Temuco 4780000, Chile; 3 Facultad de Ciencias de la Rehabilitación y Calidad de Vida, Escuela de Kinesiología, Universidad San Sebastián, Lago Panguipulli 1390, Puerto Montt 5501842, Chile; guido.contreras@uss.cl; 4 Exercise and Rehabilitation Sciences Institute, School of Physical Therapy, Faculty of Rehabilitation Sciences, Universidad Andres Bello, Santiago 7591538, Chile; 5 Departamento de Ciencias de la Educación, Universidad del Bio-Bio, Chillán 3800708, Chile; chernandez@ubiobio.cl; 6 Department of Health Rehabilitation Sciences, Universidad del Bio-Bio, Chillán 4081112, Chile; 7 Department of Physical Education, Sport and Recreation, Universidad de La Frontera, Temuco 4811230, Chile

**Keywords:** health-related quality of life, KIDSCREEN-10, screen time, sleep duration, cognitive flexibility, mediation analysis

## Abstract

Executive functions (EFs) are key cognitive processes for behaviour. However, there is little information about interaction with the dimensions of health-related quality of life (HRQoL), therefore the objective of this study was to analyse the association between lifestyle habits (physical activity and screen time), sleep, HRQoL and EFs in children and adolescents. Specifically, this study aimed to identify the extent to which perceived well-being dimensions are associated with EFs and to determine the potential mediating role of HRQoL in the relationships between lifestyle habits and these cognitive domains, examining whether these direct and indirect pathways remain robust after adjusting for gender and age. A total of 943 children and adolescents (51.3% female) aged 10–17 years participated. Lifestyle parameters (PA Krece Plus, sleep duration and KIDSCREEN-10 questionnaire) and EFs (CogniFit neurocognitive assessment battery) were evaluated. The analysis of the individual KIDSCREEN-10 items revealed that perception of school performance presented the most consistent association with EFs, being positively related to attention (*b* = 16.39, *p* = 0.018), cognitive flexibility *(b* = 30.65, *p* = 0.005), inhibition (*b* = 24.66, *p* = 0.022), and working Memory (*b* = 42.33, *p* < 0.001). Furthermore, parental fairness reported a significant association for three out of four domains: attention (*b* = 13.89, *p* = 0.006), flexibility (*b* = 22.93, *p* = 0.003), and working Memory (*b* = 25.42, *p* < 0.001). Having enough time for self was also significantly related to attention performance (*b* = 12.60, *p* = 0.026). Regarding lifestyle habits, the composite lifestyle index (PA + ST) showed the most consistent positive association across all cognitive domains, while sleep duration was inversely associated with attention, cognitive flexibility, and working Memory. The mediation analysis revealed that global HRQoL significantly mediated the relationship between lifestyle habits and executive functions, accounting for 9.55% of the total effect on attention, 5.45% on cognitive flexibility, and 4.14% on working memory, whereas no mediation was observed for inhibition. In conclusion personal time, parental fairness, and school adjustment were positively associated with EFs. HRQoL and physical activity levels also showed consistent links with all EFs, whereas sleep duration was inversely related. Furthermore, mediation analysis revealed that global HRQoL acts as a critical indirect pathway, explaining a significant proportion of the lifestyle habits’ total effect on attention, cognitive flexibility, and working memory. Overall, these findings highlight the multifactorial and interrelated mechanisms shaping executive functioning in children and adolescents.

## 1. Introduction

Executive functions (EFs) are key cognitive processes for behaviour (Turnes & Piacentini, 2025). Inhibitory control, working memory and cognitive flexibility are among them (Diamond, 2013) and are related as predictors of academic performance, psychosocial adaptation, emotional regulation, and decision-making. It has been determined that well-established EFs are related to better school results and social integration, while their deficit is related to unfavourable conditions in mental health and daily functioning (Lobato-Ruiz et al., 2025). On the other hand, there are fundamental variables such as personal time, parental equity, school adjustment, and levels of physical activity (PA), which interact with EFs and health-related quality of life (HRQoL) in a complex and multidimensional scenario that encompasses physical, emotional, and social well-being (Subramanyam et al., 2024).

Aspects such as autonomy in personal time, flexible management, and the perception of parental equity are beneficial references associated with self-regulation, stress reduction, and psychological well-being (Mikhaylova et al., 2023; Pandey et al., 2018). It has been found that interventions in self-regulation—including autonomy—generate moderate improvements in EFs with fewer incidents of stressful situations and HRQoL (Pandey et al., 2018). In the same context, parental stress has been linked to perceptions of unfair treatment, which has been shown to have a negative impact on executive functions and performance in daily life activities. Therefore, executive deficits could be correlated with parental conflicts (Lobato-Ruiz et al., 2025), and, conversely, there are parental factors that enhance cognitive resources, reducing risks such as emotional dysregulation and supporting indicators of quality of life. Among these variables are family dynamics and mental health (Subramanyam et al., 2024).

In educational contexts, peer and teacher relationships and issues such as a sense of belonging and academic performance are relevant factors that can generate demands or reinforcements of EFs (Donenfeld et al., 2026; Selman & Dilworth-Bart, 2024). It has been established that, although moderate, there are relationships with routine management, such as the presence of consistent schedules, which increase self-regulatory skills and levels of connection, interest, and active academic interaction (Donenfeld et al., 2026). The presence of successful adjustments of these variables is related to emotional well-being and a low presence of mental health problems, which indirectly strengthen EFs (Ramos-Monsivais et al., 2024). In high-risk environments, routines mitigate adversity, fostering empathy and reducing behavioural problems (Selman & Dilworth-Bart, 2024). On the other hand, in the context of PA levels and screen time (ST), both variables can be associated with HRQoL (Guzmán-Muñoz et al., 2025; Toth et al., 2025).

Excessive ST is associated with poorer EFs, reduced attention span, and impulsivity (Toth et al., 2025). Conversely, reduced ST is associated with better inhibitory control and a lower risk of obesity (Liu et al., 2022), while more time spent in front of screens has been linked to negative impacts on EFs and sleep (Xu et al., 2025). In contrast, greater frequency of PA has favourable impacts on inhibitory control and working memory (Alhwaiti, 2025), reflecting that active lifestyles are linked to better cognition (Mora-Gonzalez et al., 2025) and that good cardiorespiratory fitness is associated with better EFs (García-Alonso et al., 2025), showing that initiating actions such as active school breaks can boost cognitive benefits, thereby reducing the negative consequences of sedentary lifestyles. The above background information shows that the study of EFs in children and adolescents involves multifactorial variables where personal time, parental equity, school adjustment and levels of PA emerge as key aspects, and their links not only have interactions with cognitive, emotional and social development but also represent an area of interest for parents and educators in terms of promoting equitable and stimulating environments that enhance self-regulation and reduce risks such as stress or sedentary lifestyles.

On the other hand, this diagnostic study is closely related to two educational policies in Chile: firstly, the “60 min” of physical activity law, which implies that schoolchildren actively move for one hour per day, and law 21.801, which prohibits the use of cell phones. These laws began to operate in 2026 and can be beneficial for children and adolescents, which should be evidenced through future studies, given the reality that exists in the Chilean case at present, as evidenced in this research. On the other hand, understanding these interactions is important for promoting effective interventions to improve the health, HRQoL, and overall well-being of the younger generations. Therefore, the objective of this study was to analyse the association between lifestyle habits (physical activity and screen time), sleep, HRQoL and EFs in children and adolescents. Specifically, this study aimed to identify the extent to which perceived well-being dimensions are associated with EFs and to determine the potential mediating role of HRQoL in the relationships between lifestyle habits and these cognitive domains, examining whether these direct and indirect pathways remain robust after adjusting for gender and age.

## 2. Materials and Methods

### 2.1. Participants

The present study employed a quantitative, cross-sectional, and descriptive–associative design. A total of 943 Chilean children and adolescents aged 10 to 17 years (mean age: 13.68 ± 1.65 years) from Temuco, Chile, participated in this study (male, n = 461; female, n = 482). The sample was intentional and non-probabilistic. The students belonged to different municipal and subsidized schools in Temuco and Padre Las Casas, Araucanía Region, Chile.

To guarantee sufficient statistical power and ensure the representative nature of the sample for the multi-variable linear regression and mediation frameworks, a prospective a priori sample size calculation was performed using the G*Power software (version 3.1.9.7) (Faul et al., 2007). To rigorously detect even minor or subtle behavioural and psychological interactions without relying on chance, the statistical parameters for an F-test were set under highly stringent criteria: a small anticipated effect size (f^2^ = 0.025), a strict significance alpha level of 0.05 (alpha = 0.05), and an exceptionally high statistical power of 95% (1 − beta = 0.95), accounting for the 3 total predictors included in the model. Based on these mathematical configurations, a minimum sample size of 691 participants was established as the mandatory threshold to avoid type II errors and ensure stable parameter estimations. Therefore, the final enrolment of 943 adolescents substantially exceeded this demanding baseline, providing an optimal and highly sensitive statistical framework to evaluate the structural pathways linking lifestyle, sleep duration, HRQoL and EFs.

The inclusion criteria were as follows: (i) participants had to be enrolled in school and (ii) be aged between 10 and 17 years. The exclusion criteria included (i) any medical contraindications that would prevent normal performance in the assessments and (ii) absence during the assessment period.

This research adhered to the principles outlined in the Declaration of Helsinki (2013) and was approved by the Ethics Committee of Universidad Autónoma de Chile (approval number: CEC 13-25). Participation in the study required signed assent from the schoolchildren themselves, as well as informed consent from their parents or guardians.

### 2.2. Main Outcomes

#### 2.2.1. Physical Activity Levels and Screen Time

The child’s lifestyle was also evaluated by the PA Krece Plus test (Majem et al., 2003), a quick questionnaire that classifies lifestyle according to the average hours spent watching television or playing video games (screen time, ST) daily and physical activity (PA) after school hours per week. The classification is made according to the number of hours for each item. To ensure the index is logically interpretable, where a higher total score reflects a healthier lifestyle, screen time hours were reverse-coded as follows: 5 points: <1 h/day, 4 points: 1 h/day, 3 points: 2 h/day, 2 points: 3 h/day, 1 point: 4 or more hours/day. Conversely, points for PA were assigned directly (higher frequency resulting in higher points).

The total points are added up, and the person is accordingly classified as having either a good lifestyle (male ≥ 9 h, female ≥ 8 h), a regular lifestyle (male 6–8 h, female 5–7 h), or a bad lifestyle (male ≤ 5 h, female ≤ 4 h). The questionnaires were completed individually by the adolescents in the presence of researchers.

#### 2.2.2. Executive Functions

To evaluate EFs, including inhibition, working memory, cognitive flexibility, and attention, the CogniFit neurocognitive assessment battery (San Francisco, CA, USA) was used (Tapia et al., 2022). This 40 min assessment provides both a general cognitive score and specific scores for EFs. The CogniFit battery has been reported to exhibit good reliability and has been successfully used with school-aged children (Reina-Reina et al., 2023).

The neuropsychological test was administered online and required approximately 30 to 40 min to complete. At the conclusion of the assessment, a comprehensive results report was automatically generated, detailing the user’s neurocognitive profile.

#### 2.2.3. Health-Related Quality of Life

Health-related quality of life (HRQoL) for participants was evaluated using the KIDSCREEN-10 questionnaire. KIDSCREEN-10 is a validated and widely used tool designed to monitor global HRQoL in children and adolescents aged 8 to 18 years. It comprises ten items, which include the following questions: (1) Have you felt fit and well? (2) Have you felt full of energy? (3) Have you felt sad? (4) Have you felt lonely? (5) Have you had enough time for yourself? (6) Have you been able to do the things that you want to do in your free time? (7) Have your parent(s) treated you fairly? (8) Have you had fun with your friends? (9) Have you got on well at school? (10) Have you been able to pay attention? (Ravens-Sieberer et al., 2010).

Each item is answered on a five-point Likert scale, reflecting the frequency of a specific behaviour or feeling (1 = never, 2 = almost never, 3 = sometimes, 4 = almost always, 5 = always) or the intensity of an attitude (1 = not at all, 2 = slightly, 3 = moderately, 4 = very, 5 = extremely). Responses to negatively formulated items (questions 3 and 4) were reverse-scored on a scale from 1 to 5. The raw scores were used for analysis, with higher values indicating better HRQoL.

#### 2.2.4. Sleep Duration

Sleep duration was assessed by self-report. Participants were asked to indicate the average number of hours of sleep they typically obtained per night on school days. This method has been widely used in epidemiological studies with children and adolescents to estimate habitual sleep patterns. The responses were recorded in hours and used as a continuous variable in the analyses.

### 2.3. Analysis Procedure

The normality of the data distribution was assessed using the Kolmogorov–Smirnov test (appropriate for sample sizes > 50). Despite some variables showing a non-normal distribution, parametric tests (*t*-test and linear regression) were employed based on the Central Limit Theorem, as the large sample size ensures the robustness of parametric estimators. Additionally, the absence of multicollinearity in the regression models was confirmed using the variance inflation factor. Data analysis was performed using IBM SPSS Statistics (version 23.0). Descriptive statistics were recorded, including means and standard deviations. A *t*-test was conducted to examine gender-based differences across all study variables. To evaluate the association of lifestyle, well-being and EFs, hierarchical linear regression models were implemented. In Model 1, the lifestyle index (PA + ST), sleep duration, and HRQoL were included as independent predictors. Model 2 adjusted for gender and age as covariates to test the robustness of the observed associations. Additionally, separate regression models were conducted using individual KIDSCREEN-10 items to identify specific associations of EFs. Statistical significance was set at *p* < 0.05, and 95% confidence intervals (CIs) were reported for all estimates.

A mediation analysis was conducted using macro/interface process v. 3.3 for SPSS v. 23, and the bootstrapping method was used with a resampling rate of 5000 (Preacher & Hayes, 2004). Lifestyle (PA and ST) was entered as the independent variable (X), HRQoL as the mediator (M), and executive functions as the dependent variable (Y). Statistical significance for indirect effects was determined using a non-parametric bootstrapping procedure. A 95% confidence interval (CI) that did not include zero was considered evidence of a significant mediation effect. Additionally, the proportion of the total effect explained by the mediator was calculated using the formula (ab/c) × 100, where ab represents the indirect effect, and c represents the total effect. All models were adjusted for gender and age as covariates (Preacher & Hayes, 2004).

## 3. Results

Table 1 shows the descriptive statistics and gender differences analysis in lifestyle and cognitive variables. Descriptive analysis and *t*-test results revealed significant gender-based differences across several lifestyle and cognitive domains. With regard to lifestyle, boys reported significantly higher daily ST (*p* < 0.001) and more hours of PA per week (*p* < 0.001) compared to girls. HRQoL was also significantly higher in boys (28.44 ± 6.19 vs. 23.95 ± 6.34, *p* < 0.001) than in girls. In terms of cognitive performance, boys outperformed girls in attention (*p* = 0.008), cognitive flexibility (*p* = 0.003), and working memory (*p* = 0.003). No significant differences were observed for age, sleep duration, the combined lifestyle index, or inhibitory control (*p* > 0.05).

The initial analysis (Model 1, Table 2) examined the relationship of lifestyle and HRQoL with EFs. Subsequently, these associations were tested for robustness by adjusting for gender and age (Model 2). In Model 1, the variables accounted for a significant amount of variance in attention scores. The composite lifestyle index (*b* = 0.12, *p* = 0.001) and global HRQoL (*b* = 0.15, *p* < 0.001) showed significant positive associations with attention performance. Conversely, sleep duration displayed a significant inverse relationship (*b* = −0.15, *p* < 0.001). Upon adjusting for gender and age in Model 2, all primary indicators remained robustly associated with attention. Regarding cognitive flexibility, Model 1 revealed that the composite lifestyle index was the indicator with the strongest positive link (*b* = 0.20, *p* < 0.001), followed by global HRQoL (*b* = 0.12, *p* = 0.002). Sleep duration maintained a significant inverse association (*b* = −0.13, *p* = 0.001). For inhibition, in Model 1, the composite lifestyle index emerged as the only indicator significantly associated with inhibition (*b* = 0.22, *p* < 0.001). This pattern was confirmed in Model 2; when gender and age were integrated into the model, only the lifestyle index retained its significant positive association (*b* = 0.21, *p* < 0.001). Finally, the analysis for working memory in Model 1 showed that the composite lifestyle index possessed the strongest positive association (*b* = 0.23, *p* < 0.001), alongside a positive link for global HRQoL (*b* = 0.10, *p* = 0.007) and a significant inverse association for sleep duration (*b* = −0.10, *p* = 0.006). After adjusting for sociodemographic factors in Model 2, these indicators remained significant explanatory factors for working memory.

The analysis of the individual KIDSCREEN-10 items (Table 3) revealed that perception of school performance (Item 9) presented the most consistent association with EFs, being positively related to attention (*b* = 16.39, *p* = 0.018), cognitive flexibility (*b* = 30.65, *p* = 0.005), inhibition (*b* = 24.66, *p* = 0.022), and working memory (*b* = 42.33, *p* < 0.001). Furthermore, parental fairness (Item 7) reported a significant association for three out of four domains: attention (*b* = 13.89, *p* = 0.006), flexibility (*b* = 22.93, *p* = 0.003), and working memory (*b* = 25.42, *p* < 0.001). Having enough time for self (Item 5) was also significantly related to attention performance (*b* = 12.60, *p* = 0.026).

### Mediation Analysis

**Figure 1:**
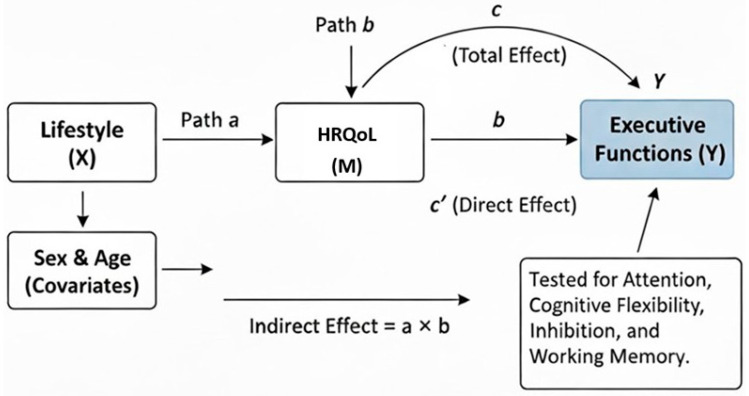
Mediation model of health-related quality of life (M) in the relationship between lifestyle (X) and executive functions (Y), adjusted for gender and age. Note: Path a represents the effect of X on M; Path b represents the effect of M on Y; Path c’ represents the direct effect of X on Y.

To examine whether global HRQoL explains the indirect mechanisms linking the composite lifestyle index (aggregating PA and screen time) to specific EFs, parallel mediation analyses were conducted, adjusting for gender and age as covariates (Figure 1, Table 4). The analysis revealed a significant positive association between lifestyle and global HRQoL across all models (Paths a:b = 0.35, SE = 0.11, *t* = 3.22, *p* = 0.001). Regarding the EF outcomes (Path b), global HRQoL exhibited significant positive relationships with attention, cognitive flexibility, and working memory. In contrast, the association between global HRQoL and inhibition did not reach statistical significance. Furthermore, significant indirect effects via global HRQoL were confirmed for attention (indirect effect = 0.93, 95% Boot CI [0.23, 1.85]), cognitive flexibility (indirect effect = 1.19, 95% Boot CI [0.17, 2.59]), and working memory (indirect effect = 0.90, 95% Boot CI [0.06, 2.09]). Based on these pathways, global HRQoL accounted for 9.55%, 5.45%, and 4.14% of the total effect linking lifestyle habits to attention, cognitive flexibility, and working memory, respectively. Conversely, no statistical mediation framework was observed for the inhibition domain, as its bootstrapping confidence interval crossed zero (indirect effect = 0.94, 95% Boot CI [−0.01, 2.21]).

## 4. Discussion

The objective of this study was to analyse the association between lifestyle habits (PA and screen time), sleep duration, HRQoL, and cognitive performance in children and adolescents. Specifically, the study aimed to identify the extent to which perceived well-being dimensions (KIDSCREEN-10) are associated with attention and executive functions and to determine the potential mediating role of HRQoL in the relationships between lifestyle habits and these cognitive domains, examining whether these direct and indirect pathways remain robust after adjusting for gender and age.

The main findings of this investigation were as follows: (i) personal time, parental fairness, and school adjustment were associated with EFs in children and adolescents; (ii) HRQoL was linked to all EFs; (iii) PA levels were related to all EFs; and (iv) sleep duration was linked inversely to EFs.

In the present study, our exploratory item-level analysis revealed that specific proxy indicators of perceived well-being from the global HRQoL, such as personal time, parental fairness, and school adjustment, were significantly associated with EF domains in children and adolescents. This aligns with prior research on school-age children and adolescents that found that children’s parenting behaviours and EF skills were positively linked (Sosic-Vasic et al., 2017). Aspects such as metacognitions and inhibitory self-control seem more related to less-sensitive parenting from the mother and harsher parenting from the father—therefore, parenting may play a role in child EFs (Lucassen et al., 2015). Hughes and Devine showed in a longitudinal analysis that a differentiated view of parental influence, negative parent–child interactions, and parental scaffolding demonstrated unique and specific associations with child EFs (Hughes & Devine, 2019). Similarly, the study by Devine et al. (Devine et al., 2016) highlighted that EFs mediated the associations between parental scaffolding and negative parent–child interactions and students’ academic ability. These findings are supported by another study by Rungsattatharm et al., in which the authors observed that positive parenting (4 and 6 years old) was related to better self-regulatory efficacy and fewer behavioural problems at age 9, mediated by reduced EF problems (Rungsattatharm et al., 2025). Another study concluded that parental behaviours during the early years of school-age children can influence the development of EFs (Valcan et al., 2018). Likewise, a systematic review reported that parent and teacher behaviour was linked with EFs in early and middle childhood (Koşkulu-Sancar et al., 2023), while a mediation model found that higher levels of engagement in educational ST were related to fewer EF problems, which in turn were associated with better school adjustment (Kim & Tsethlikai, 2025). Moreover, it has been indicated that EF domains track meaningfully with emotional and behavioural adjustment indices (Cassidy, 2016). Chi et al. suggested that EFs can predict academic adjustment in children (Chi et al., 2018). These findings underline that having better EFs is an important factor since they can also be positively related to perceived social support and social adjustment (Chen et al., 2024). In addition, a longitudinal study reported that EFs and learning-related behaviours are necessary to support efforts to promote school adjustment (Sasser et al., 2015).

In the present study, HRQoL was linked to all EFs. The fact that HRQoL and EFs have a complex interactive relationship has been previously evidenced (Laera et al., 2023). Adding to the above, a study carried out by Huang et al. claimed that there is evidence that high levels of EFs and better HRQoL are closely linked (Huang et al., 2020). Our findings are supported by research by Goethals et al. (Goethals et al., 2021), in which the authors observed that there existed a link between low EFs and poorer HRQoL. In addition, research has proved that a better HRQoL during childhood is related to better inhibition and cognitive flexibility (Huang et al., 2020). Similarly, after a two-year follow-up investigation, Cushman et al. (Cushman et al., 2022) indicated that subjects with poorer EFs had better mental and physical HRQoL. These findings underline the notion that HRQoL could affect cognitive functions (Lemes et al., 2021). Another study found that in a bidirectional way, metacognition predicted the social and school-related dimensions of HRQoL in schoolchildren (Hammud et al., 2023).

PA levels were related to all EFs. Our findings are supported by the study by Zeng et al., which showed that PA was positively linked to better EFs at the school stage (Zeng et al., 2022). In addition to the above, previous evidence has shown that low PA in school could have a negative association with EFs (Cai et al., 2025). Also, research has proven that better PA patterns are linked to higher scores on inhibitory control and working memory tasks (García-Alonso et al., 2025). Another study found that students meeting the 24 h movement requirement presented better global performance and cognitive flexibility (Zeng et al., 2022). Similarly, the study conducted by Contreras et al. on Chilean schoolchildren reported that better PA levels were positively related to better working memory, cognitive flexibility, and inhibition (Contreras-Osorio et al., 2022). Furthermore, it has been shown that systematic PA leads to improvements in physical fitness and may support cognitive skills in youth (Muntaner-Mas et al., 2022). In a longitudinal analysis, Liu et al. (2024) found that the substitution of ST with PA may be a beneficial procedure in enhancing EFs. Another study reported a long-term link between PA and EFs (Galle et al., 2023). Additionally, a further study highlighted that being physically active at any time in adulthood was related to a better later-life cognitive state (James et al., 2023). In a school setting, for instance, demonstrating that allocating time to PA does not negatively influence cognitive and academic performance is a practically important finding given the wealth of broader benefits to students (Northey et al., 2025).

Several researchers have affirmed that PA can influence brain function through the modulatory effects of brain-derived neurotrophic factor (BDNF) and hormones and metabolites of the muscle–brain axis, such as irisin, lactate, cathepsin B, kynurenine, and insulin-like growth factor-1 (IGF-1) (Dadkhah et al., 2023; Nay et al., 2021). In addition, we found that sleep duration was inversely linked to EFs. In this regard, and contrary to our results, it has been indicated that seven hours of sleep per day is associated with the highest cognitive performance, which decreases for every hour below and above this sleep duration (Tai et al., 2022). Another study indicated that suboptimal sleep was independently linked with worse cognitive performance; short sleep was also associated with faster cognitive decline (Bloomberg et al., 2023). This study only considers self-reported sleep hours, so our results differ from some previous studies. In the future, we plan to use accelerometry to clarify the relationship between sleep and EFs. In addition, while our results differ in the sleep variable from what has been reported in the literature, one study indicates that a linear increase in sleep duration has a small negative effect on cognitive skills (i.e., reaction time and visual memory), but the true association might be non-linear (Henry et al., 2019). The Avena study conducted on young people did not find a significant association between sleep duration and cognitive performance in adolescent females (Ortega et al., 2010). In this sense, another study showed that sleep duration was not associated with sustained attention and memory (Astill et al., 2012).

While these observed co-variations highlight a significant statistical link between a supportive lifestyle index, perceived well-being, and EFs, these cross-sectional findings must be interpreted with caution before drawing direct educational or clinical implications. Because the present study design cannot establish chronological precedence or causal direction, we cannot conclude that modifying screen time or sleep duration will directly trigger an improvement in EFs. Instead, these data suggest that lifestyle habits and perceived HRQoL form a complex, interrelated network that co-varies with cognitive performance. Consequently, while these indicators are valuable for identifying at-risk school profiles, broader educational or health policies should wait for longitudinal and experimental confirmation to establish the true directionality and practical impact of these mechanisms.

### Strengths and Limitations

Despite its contributions, this study is not without limitations; primarily, its cross-sectional design precludes establishing definitive causal relationships, making future longitudinal research mandatory to confirm the proposed statistical mechanisms. Additionally, relying on self-reported questionnaires for lifestyle habits (Krece Plus) and health-related quality of life (KIDSCREEN-10) introduces potential risks of recall and social desirability bias, while the use of a non-probabilistic convenience sample restricts the generalizability of the findings to the national population.

Regarding the methodology, evaluating each KIDSCREEN-10 question individually could increase the risk of finding accidental or false associations due to the high number of statistical tests performed; however, these item-level analyses were strictly treated as exploratory data to inform the global models. Conversely, a major strength of this study is its robust sample size, which provided adequate statistical power to control for age and gender. Furthermore, unlike many epidemiological studies that rely on subjective assessments, this research utilized CogniFit, a highly standardized and objective computer-based neurocognitive battery that allows for a direct and precise quantification of distinct executive domains (attention, cognitive flexibility, inhibition, and working memory) in children and adolescents.

## 5. Conclusions

In conclusion, this study demonstrates a significant cross-sectional association between lifestyle habits, HRQoL, and EFs in children and adolescents. The exploratory analysis indicates that individual dimensions of perceived well-being, particularly personal time, parental fairness, and school adjustment, co-vary positively with EFs even after statistical control for gender and age. Crucially, the mediation analysis reveals that global HRQoL acts as a significant statistical mechanism that partially underlies the relationships between a composite lifestyle index and the specific domains of attention, cognitive flexibility, and working memory. These findings underscore the importance of examining HRQoL not as an isolated outcome, but as an interconnected factor linked to both daily habits and executive functioning. However, given the cross-sectional nature of this evidence, these results should be interpreted as safe indicators of statistical co-variation rather than causal direction, highlighting the need for future longitudinal designs to confirm these pathways.

## Figures and Tables

**Table 1:** Descriptive statistics and gender differences in lifestyle and cognitive variables.

Variable	Boys (n = 461)	Girls (n = 482)	Total (N = 943)	*p*-Value	F-Value
Age (years)	13.83 ± 2.02	13.82 ± 2.00	13.82 ± 2.01	0.932	0.01
Screen Time (h/day)	3.38 ± 1.64	2.99 ± 1.61	3.18 ± 1.63	0.000	13.88
Physical Activity (h/week)	2.72 ± 1.85	2.18 ± 1.65	2.44 ± 1.77	0.000	21.38
Lifestyle (PA + ST)	4.57 ± 2.34	4.39 ± 2.20	4.47 ± 2.27	0.252	1.31
Sleep Duration (h/day)	8.01 ± 1.47	7.83 ± 1.53	7.92 ± 1.50	0.070	3.29
Health Related to Quality of Life	28.44 ± 6.19	23.95 ± 6.34	26.15 ± 6.65	0.000	121.43
Cognitive Area: Attention	428.06 ± 150.66	399.54 ± 158.39	413.49 ± 155.22	0.008	7.02
EFs: Cognitive Flexibility	403.40 ± 237.88	353.04 ± 239.80	377.73 ± 240.04	0.003	9.15
EFs: Inhibition	312.28 ± 225.61	325.79 ± 248.16	319.17 ± 237.32	0.415	0.67
EFs: Working Memory	256.17 ± 224.85	213.10 ± 196.79	234.22 ± 211.98	0.003	8.58

Note. Data presented as mean ± standard deviation. EFs = executive functions; PA = physical activity; ST = screen time. *p* < 0.05 indicates statistical significance.

**Table 2:** Association of lifestyle and HRQoL with executive functions.

0		*b*	95%CI		SE	*t*	*p*-Value
Attention							
Model 1	Lifestyle (PA + ST)	8.49	3.36	13.63	2.62	3.25	0.001
	Sleep (h)	−15.46	−22.98	−7.93	3.83	−4.03	*p* < 0.001
	HRQoL	3.45	1.70	5.20	0.89	3.88	*p* < 0.001
Model 2	Lifestyle (PA + ST)	8.40	3.24	13.57	2.63	3.19	0.001
	Sleep (h)	−15.44	−23.06	−7.83	3.88	−3.98	*p* < 0.001
	HRQoL	2.93	1.10	4.77	0.93	3.15	0.002
	Cognitive Flexibility						
Model 1	Lifestyle (PA + ST)	21.74	13.76	29.73	4.07	5.35	*p* < 0.001
	Sleep (h)	−20.53	−32.25	−8.81	5.97	−3.44	0.001
	HRQoL	4.33	1.60	7.05	1.39	3.12	0.002
Model 2	Lifestyle (PA + ST)	20.34	12.36	28.32	4.06	5.00	0.000
	Sleep (h)	−18.05	−29.83	−6.26	6.00	−3.01	0.003
	HRQoL	3.73	0.90	6.55	1.44	2.59	0.010
	Inhibition						
Model 1	Lifestyle (PA + ST)	23.63	15.78	31.48	4.00	5.91	*p* < 0.001
	Sleep (h)	−6.34	−17.87	5.20	5.87	−1.08	0.281
	HRQoL	1.86	−0.82	4.54	1.36	1.36	0.173
Model 2	Lifestyle (PA + ST)	22.36	14.48	30.24	4.02	5.57	0.000
	Sleep (h)	−3.74	−15.39	7.91	5.93	−0.63	0.528
	HRQoL	2.59	−0.21	5.39	1.42	1.82	0.069
	Working Memory						
Model 1	Lifestyle (PA + ST)	22.37	15.33	29.41	3.58	6.24	*p* < 0.001
	Sleep (h)	−14.55	−24.89	−4.22	5.26	−2.77	0.006
	HRQoL	3.28	0.88	5.68	1.22	2.68	0.007
Model 2	Lifestyle (PA + ST)	20.41	13.45	27.37	3.54	5.76	*p* < 0.001
	Sleep (h)	−11.00	−21.28	−0.73	5.23	−2.10	0.036
	HRQoL	2.75	0.28	5.21	1.26	2.18	0.029

Note: *b*: unstandardized regression coefficient; 95% CI: 95% confidence interval for *b*; SE: standard error; *t*: *t*-test value. Model 1: unadjusted model. Model 2: adjusted for age and gender. PA: physical activity, ST: screen time; HRQoL: health-related quality of life.

**Table 3:** Personal time, parental fairness, and school adjustment KIDSCREEN-10 dimensions related to executive functions.

KIDSCREEN-10 Item	Attention *b* (95% CI)	*p*-Value	Cognitive Flexibility *b* (95% CI)	*p*-Value
1. Physical well-being	2.57 (−9.34, 14.47)	0.672	9.26 (−9.19, 27.70)	0.325
2. Energy levels	−7.63 (−18.75, 3.50)	0.179	−12.82 (−30.07, 4.43)	0.145
3. Sadness	−9.14 (−20.89, 2.61)	0.127	−4.07 (−22.36, 14.21)	0.662
4. Loneliness	6.77 (−4.28, 17.81)	0.229	3.99 (−13.11, 21.08)	0.647
5. Time for self	12.60 (1.53, 23.66)	0.026	14.17 (−2.95, 31.30)	0.105
6. Free time activities	−2.83 (−13.78, 8.11)	0.612	−12.28 (−29.24, 4.67)	0.155
7. Fairness (Parents)	13.89 (4.03, 23.75)	0.006	22.93 (7.65, 38.21)	0.003
8. Fun with friends	−3.87 (−14.98, 7.23)	0.494	−2.90 (−20.17, 14.38)	0.742
9. School performance	16.39 (2.81, 29.96)	0.018	30.65 (9.43, 51.86)	0.005
10. Attention capacity	5.21 (−8.17, 18.58)	0.445	9.20 (−11.61, 30.02)	0.386
	**Inhibition** ***b* (95% CI)**	***p*-Value**	**Working Memory** ***b* (95% CI)**	***p*-Value**
1. Physical well-being	7.84 (−10.51, 26.19)	0.402	0.95 (−15.22, 17.13)	0.908
2. Energy levels	−14.34 (−31.50, 2.83)	0.101	−9.79 (−24.92, 5.34)	0.204
3. Sadness	−4.44 (−22.63, 13.75)	0.632	−8.06 (−24.09, 7.98)	0.324
4. Loneliness	9.81 (−7.20, 26.82)	0.258	3.15 (−11.84, 18.14)	0.68
5. Time for self	15.01 (−2.03, 32.05)	0.084	4.50 (−10.52, 19.52)	0.557
6. Free time activities	−15.26 (−32.13, 1.61)	0.076	−14.68 (−29.55, 0.19)	0.053
7. Fairness (Parents)	14.95 (−0.26, 30.15)	0.054	25.42 (12.02, 38.83)	<0.001
8. Fun with friends	−1.51 (−18.70, 15.68)	0.863	−4.03 (−19.18, 11.12)	0.602
9. School performance	24.66 (3.55, 45.77)	0.022	42.33 (23.73, 60.94)	<0.001
10. Attention capacity	12.16 (−8.55, 32.87)	0.249	0.22 (−18.04, 18.47)	0.981

Notes: *b*: unstandardized coefficient; 95% CI: 95% confidence interval for *b*. *p* < 0.05 indicates statistical significance. Items from the KIDSCREEN-10 index: Physical well-being: Have you felt fit and well? Energy levels: Have you felt full of energy? Sadness: Have you felt sad? Loneliness: Have you felt lonely? Time for self: Have you had enough time for yourself? Free time activities: Have you been able to do the things that you want to do in your free time? Fairness (parents): Have your parent(s) treated you fairly? Fun with friends: Have you had fun with your friends? School performance: Have you got on well at school? Attention capacity: Have you been able to pay attention?

**Table 4:** Mediation analysis of the effect of lifestyle on executive functions through HRQoL.

Dependent Variable (Y) and Mediation Paths	Coeff (*b*)	SE	*t*	*p*-Value	95% CI	Mediation Proportion (%)
**Attention**						
Path a:	0.35	0.11	3.22	0.001	[0.14, 0.56]	—
Path b:	2.70	0.93	2.91	0.004	[0.88, 4.52]	—
Direct Effect (c’)	8.84	2.64	3.34	<0.001	[3.65, 14.03]	—
Total Effect (c)	9.77	2.64	3.70	<0.001	[4.59, 14.95]	—
Indirect Effect (a × b)	0.93	0.42	—	—	[0.23, 1.85]	9.55%
**Cognitive Flexibility**						
Path a:	0.35	0.11	3.22	0.001	[0.13, 0.56]	—
Path b:	3.46	1.43	2.43	0.015	[0.66, 6.26]	—
Direct Effect (c’)	20.69	4.05	5.10	<0.0001	[12.73, 28.65]	—
Total Effect (c)	21.89	4.04	5.42	<0.0001	[13.96, 29.82]	—
Indirect Effect (a × b)	1.19	0.62	—	—	[0.17, 2.59]	5.45%
**Inhibition**						
Path a:	0.35	0.11	3.22	0.001	[0.13, 0.56]	—
Path b:	2.74	1.4	1.96	0.050	[−0.01, 5.48]	—
Direct Effect (c’)	23.02	3.98	5.79	<0.0001	[15.21, 30.83]	—
Total Effect (c)	23.97	3.96	6.06	<0.0001	[16.20, 31.73]	—
Indirect Effect (a × b)	0.94	0.58	—	—	[−0.01, 2.21]	No mediation
**Working Memory**						
Path a:	0.35	0.11	3.22	0.001	[0.13, 0.56]	—
Path b:	2.62	1.24	2.12	0.035	[0.19, 5.05]	—
Direct Effect (c’)	20.94	3.52	5.95	<0.0001	[14.03, 27.86]	—
Total Effect (c)	21.85	3.5	6.23	<0.0001	[14.97, 28.73]	—
Indirect Effect (a × b)	0.9	0.52	—	—	[0.06, 2.09]	4.14%

Note: Path coefficients are unstandardized (*b*). All models adjusted for gender and age.

## Data Availability

The data presented in this study are available upon request from the corresponding author (P.D.-F.). The data are not publicly available due to privacy and ethical restrictions involving school-aged participants, as per the protocols approved by the Ethics Committee of Universidad Autónoma de Chile (ACTA; No. CEC 13-25).
